# A Simplified Quantum Walk Model for Predicting Missing Links of Complex Networks

**DOI:** 10.3390/e24111547

**Published:** 2022-10-28

**Authors:** Wen Liang, Fei Yan, Abdullah M. Iliyasu, Ahmed S. Salama, Kaoru Hirota

**Affiliations:** 1 School of Computer Science and Technology, Changchun University of Science and Technology, Changchun 130022, China; 2 College of Engineering, Prince Sattam Bin Abdulaziz University, Al-Kharj 11942, Saudi Arabia; 3 School of Computing, Tokyo Institute of Technology, Yokohama 226-8502, Japan; 4 Faculty of Engineering and Technology, Future University in Egypt, Cairo 11835, Egypt; 5 School of Automation, Beijing Institute of Technology, Beijing 100081, China

**Keywords:** quantum walk, cyberphysical systems, complex networks, missing link prediction, Grover diffusion operator

## Abstract

Prediction of missing links is an important part of many applications, such as friends’ recommendations on social media, reduction of economic cost of protein functional modular mining, and implementation of accurate recommendations in the shopping platform. However, the existing algorithms for predicting missing links fall short in the accuracy and the efficiency. To ameliorate these, we propose a simplified quantum walk model whose Hilbert space dimension is only twice the number of nodes in a complex network. This property facilitates simultaneous consideration of the self-loop of each node and the common neighbour information between arbitrary pair of nodes. These effects decrease the negative effect generated by the interference effect in quantum walks while also recording the similarity between nodes and its neighbours. Consequently, the observed probability after the two-step walk is utilised to represent the score of each link as a missing link, by which extensive computations are omitted. Using the AUC index as a performance metric, the proposed model records the highest average accuracy in the prediction of missing links compared to 14 competing algorithms in nine real complex networks. Furthermore, experiments using the precision index show that our proposed model ranks in the first echelon in predicting missing links. These performances indicate the potential of our simplified quantum walk model for applications in network alignment and functional modular mining of protein–protein networks.

## 1. Introduction

Prediction of missing links, also known as link prediction, is focused on predicting missing (deleted) links or existence of possible links in future. For example, in some social media platforms, the network considers the existence of multiple common friends between a user and some strangers as justification to expand one’s circle of friends. Such a scenario is caused by effects generated by application of link prediction. In such application, the similarities between nodes are the main reference in link prediction [1,2]. In algorithms for predicting missing links for personalised recommendation [3,4], network alignment [1], broad learning [2,5], and exploration of functional modularity of protein–protein networks [6], temporal and resource costs are conserved. Meanwhile, in complex networks, missing link prediction can be classified into three categories: similarity-based algorithms, structural characteristics of network-based likelihood analysis algorithms (models), and algorithms with heuristic information.

In similarity-based algorithms for link prediction, Common Neighbour (CN) is the most intuitional information that records the number of common neighbours between two nodes in a complex network [7]. Using CN, many algorithms (also called indices) such as Jaccard index [8], Salton index [9], Sorenson index [10], etc. [2,11,12] have been designed and improved. Despite the effectiveness and simplicity of these indices, their accuracy is lean due to sensitive to structural characteristics of the network. To improve accuracy in prediction of missing links, path-based similarity algorithms and random walk-based similarity algorithms were put forward, where the length of path-based algorithms that are known to affect the prediction accuracy [13,14] are considered. Moreover, it is considered that the accuracy of local random walk-based algorithms is also sensitive to walk steps [14,15,16]. Consequently, in various types of networks, the prediction accuracy utilising path-based and random walk-based algorithms are deemed unstable.

By contrast, structural characteristics of network-based likelihood analysis algorithms (models) have exhibited high accuracy in link prediction where Bayesian analysis and network characteristics are utilised simultaneously, such as the hierarchical structure model [17], stochastic block model [18], and loop model [19]. However, these likelihood analysis-based algorithms lack practicality in link prediction due to excessive time complexity. Accordingly, heuristic information attached to an existing conventional algorithm has been considered as an innovative direction to improve existing models. For instance, since the links between similar nodes are preferentially existent in the same community, community detection is used to increase the scores of links located in the same community and improve accuracy [20]. Furthermore, since demand associated with deciding nonexistent links is one major reason for the low accuracy of the algorithms, *k*-shell is utilised to determine if there is an existent link in the original network to improve accuracy of prediction [21]. However, considering the heuristic algorithms that use community information as an instance, the number of detected communities and a network with or without obvious community structure will affect the performance of an algorithm. Consequently, prediction accuracy of existing algorithms with heuristic information is further affected by structural characteristics of various types of networks.

Based on the above analysis, the prediction accuracy for link prediction algorithms needs further improvement. Recently, the superlative performance reported from the use of quantum walks in complex network analysis has been harnessed for community detection [22], identification of significant link [23] and node [24,25], and also in similarity judgement between network graphs [26]. Exploring the reported advantages of quantum walks, this study proposes a simplified quantum walk model to predict missing links in complex networks. Our study makes two major contributions:

(i) Compared to existing quantum walk models on a complex network, the complexity and the negative effects generated by the interference effect of quantum walk are minimised in the proposed simplified quantum walk model. In contrast, since the construction method entirely depends on direct product operation and restricts by strict unitary evolution, other quantum walks on the complex network do not circumvent these effects and cannot effectively develop the structural characteristics of networks.
(ii) The proposed model can be utilised for predicting the missing links of complex networks, which has a high accuracy in two indices on link prediction, because the common neighbour and similarity between nodes are effectively contained in the evolution operator of the proposed model. Experimental validation of the proposed model indicates that, despite limiting its walk to two steps, the proposed simplified quantum walk model outperforms other random walk-based algorithms as well as global similarity-based algorithms in the prediction of missing links.

The remainder of the study is organised as follows: in Section 2, the intrigues of missing link prediction are introduced as a prelude to outlining our proposed simplified quantum walk model on complex networks in latter parts of the same section. In Section 3, the nine real complex networks and fourteen comparison algorithms used for our experiments are described while outcomes of experiments on AUC and precision indexes are also reported and discussed. Conclusions and future work are written in Section 4.

## 2. Predicting Missing Links and the Proposed Simplified Quantum Walk Model

This section first describes the definition of a link prediction problem and its evaluation criteria, and then introduces the proposed simplified quantum walk model.

### 2.1. Prediction of Missing Links in Complex Networks

**Figure 1:**
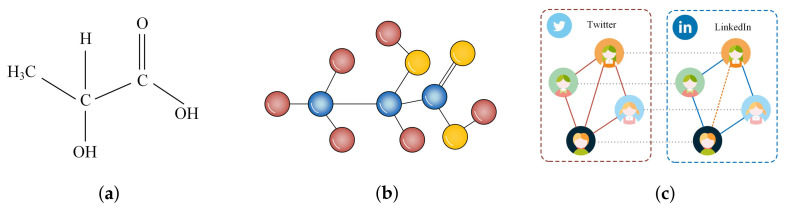
Illustration of complex network and link prediction. (**a**) structure of lactic aid; (**b**) molecular graph of lactic aid; (**c**) illustration of link prediction.

Given an undirected complex network ϱ=(V,E), *V* represents the set of nodes and *E* denotes the set of links, |V|=N and |E|=M. A simple undirected complex network represented by a lactic aid molecular is depicted in Figure 1a, and its structural graph is presented in Figure 1b. Moreover, in real life, social relationships among individuals, purchase recording among various commodities, and interaction relation between proteins can be abstracted as networks, which are similar to the form depicted in Figure 1b.

In such a network ϱ, assuming that set *E* is randomly divided into two components $E^{T}$ and $E^{P}$ based on a probability η; then, set $E^{T}$ is utilised for storing training data and $E^{P}$ is employed for recording missing links, which are links to be predicted (also called as prediction set). Two sets obtained by dividing satisfy the relation $|E^{T}|=\eta |E|\left(\eta \in \left(0,1\right)\right)$, $E^{T}\cap E^{P}=⌀$, and $E^{T}\cup E^{P}=E$. Meanwhile, for a network $\tilde{G}$ composed of *V* and $E^{T}$, i.e., $\tilde{G}=\left(V,E^{T}\right)$, the purpose of link prediction is to predict links in $E^{P}$ by scoring all links using some algorithm (model) where all links belong to a universal set $E^{U}$, $E^{T}\subseteq E^{U}$ and $E^{P}\subseteq E^{U}$. In other words, when an algorithm is utilised for predicting missing links, it scores all links in $\tilde{G}$ corresponding to a completed network, whether the link exists in $\tilde{G}$ or not. To elucidate, suppose that an individual simultaneously has social accounts on the Twitter and LinkedIn platforms, as described in Figure 1c, where solid lines between users represent the existing friend relations, and the dashed line coloured by orange between users denotes a possible link in future. It can then be surmised that the dashed line corresponds to a missing link that can be predicted by computing the similarity between two users.

Moreover, since the similarity between two nodes is regarded as an inference to link prediction [1], then the predicted result using an algorithm can be quantified by scoring to similarity between nodes. Generally speaking, the common practice is to use one major index and one minor index as a criterion to estimate the performance of an algorithm (a model) [2]. In many instances, the area under the receiver operating characteristic curve (AUC) is employed as a major index for evaluating accuracy of an algorithm in link prediction, while the precision index is used as a minor index to further compare performance in the prediction of missing links, especially when obtained AUC values using various algorithms are approximate. In the AUC index, the links in a prediction set are denoted as $E^{P}$ and those in $\left(E^{U}-E\right)$, (which denote nonexistent links in $\tilde{G}$), are randomly and independently sampled multiple times and 1 is added to the AUC value if the score of a link in $E^{P}$ is greater than score of a link in $\left(E^{U}-E\right)$. When two scores are equal, 0.5 is added to the AUC value, and the value is unchanged otherwise. Consequently, when sample times are set to *n*, the AUC index is defined as

(1) $$AUC=\frac{n_{1}+0.5n_{2}}{n},$$

where $n=n_{1}+n_{2}$. Additionally, the precision index is used to record the accurate forecast ratio in top-L links, where it is assumed that *l* links are accurately predicted. Thence, the precision index can be defined as

(2) $$P_{re}=\frac{l}{L},$$

where *L* denotes the total number of links to be evaluated, L<M. In instances where predicted outcomes of various algorithms correspond to the approximate in the AUC index, the algorithm with the greater precision value is considered as a better choice in the prediction of missing links.

### 2.2. Application of the Proposed Model in Link Prediction

Implementation of the proposed simplified quantum walk model on a complex network is built on three parts: definition of Hilbert space and state vector on the complex network, construction of evolution operator during the walking, and observation of similarity between two nodes based on the evolved result.

#### 2.2.1. Hilbert Space and State Vector on a Complex Network

**Figure 2:**
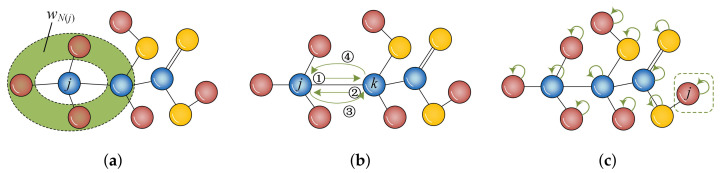
Illustration of merging idea, traceback, and self-loop in quantum walks. (**a**) merging idea; (**b**) traceback; (**c**) self-loop.

To definite the Hilbert space of our model on a complex network, the number of selectable walk directions of each node is required to be determined. Different from existing quantum walks on regular graphs, the determination and compression of the spatial dimensionality associated with the proposed model on complex networks is a challenge because each node in complex networks has an irregular number of neighbours. Considering a network $\tilde{G}=\left(V,E^{T}\right)$ as defined in Section 2.1, and assuming that the neighbours of node *j* are regarded as a whole (recorded as $w_{N(j)}$), then, for such a node, only two directions can be determined as paths of the walk, i.e., from node *j* to itself and from node *j* to its neighbours $w_{N(j)}$, respectively. In $w_{N(j)}$, N(j) denotes the neighbours of node *j* while notation $w_{N(j)}$ of merging the neighbours N(j) of node *j* as a whole is illustrated in Figure 2a, where the range coloured in green expresses neighbours of node *j* that are treated in unison, i.e., $w_{N(j)}$. Since for a node in network $\tilde{G}$, only two directions can be selected in the process of walking and there are *N* nodes in network $\tilde{G}$; therefore, the dimensionality *D* of the Hilbert space of our model on network $\tilde{G}$ is equal to twice the number of nodes in network $\tilde{G}$, i.e., D=2N. In contrast to existing studies on quantum walk models for complex network, the total dimensionality of the Hilbert space is greatly decreased in our proposed model. For example, for an identical network $\tilde{G}$, the dimensionality of Hilbert space in Szegedy’s walk is equal to $N^{2}$ [24,27], the dimensionality of Fourier quantum walk and other quantum walk achieved by direct sum operation [22,28] is equal to twice the number of links in $\tilde{G}$, i.e., $2|E^{T}|$. Consequently, less space consumption is expected from the proposed quantum walk model, especially when $\tilde{G}$ belongs to a dense network.

Meanwhile, in quantum walks, the network on which particles walk is always viewed as a quantum system, and the change of such a system is recorded and expressed by a state vector. Since, as previously observed, the dimensionality of network system $\tilde{G}$ is defined as 2N; then, the number of elements in state vector |ψ〉 is equal to 2N. As also mentioned earlier, there are two possible directions during a walk we denote as |0〉 and |1〉, respectively. Therefore, assuming that |ψ〉 is a superposition at initial time (t=0), expressed as

(3) $$|\psi \left(0\right)\rangle =\sum_{c=0}^{1}|c\rangle ⊗\sum_{j=1}^{N}\alpha_{j}\left(0\right)|j\rangle ,$$

where |j〉 expresses the orthogonal basic of node *j*, c∈{0,1}, and $\alpha_{j}\left(0\right)$ represents probability amplitude of node *j* at initial time such that the initial probability amplitude can be considered as equal superposition, then $\alpha_{j}\left(0\right)=\frac{1}{\sqrt{N}}$. Moreover, in other quantum walk models, such as Fourier walk [22], the state vector at initial time is constructed by direct sum operation of orthogonal basis of each node. By contrast, the computational form of state vector in our model has lesser time complexity.

#### 2.2.2. Construction of Evolution Operator in the Proposed Model

The construction of the evolution operator is a key step towards the prediction of missing links in the proposed simplified quantum walk model. This corresponds to a series of important steps in typical non-quantum algorithms. In the proposed model, the evolution operator incorporates both common neighborhood and connectivity information between nodes to ensure that each link of the network is predicted based on similarity characteristics between nodes. Assuming that the adjacency matrix of network $\tilde{G}$ is *A*, $A_{j,k}$ denotes the connectivity of node *j* and node *k*; then, $A_{j,k}$ is 0 if $e\left(j,k\right)\notin E^{T}$, and $A_{j,k}$ is equal to 1 if $e\left(j,k\right)\in E^{T}$. Referring to form of the Grover diffusion operator [29], each element in coin operator is defined as

(4) $$G_{j,k}^{C}=-A_{j,k}+\frac{2}{\left|\Gamma (j,k)\right|}.$$

In Equation (4), |Γ(j,k)| denotes the number of common neighbours between node *j* and *k* in $\tilde{G}$ and according to it, the full coin operator $G^{C}$ can be realised. It is trivial that the Grover coin presented in Equation (4) contains the similarity information and connectivity between an arbitrary pair of nodes.

Meanwhile, in terms of the shift operator, notations |0〉 and |1〉 often represent left and right walk directions, whereas since neighbours of node *j* are considered as a whole (as depicted in Figure 2a), the notations |0〉 and |1〉 in the proposed model denote walking along the self-loop of current node *j* in the direction of node *j*. Consequently, the shift operator in the proposed simplified model is defined as the accumulated sum of different walk direction |c〉(c=0,1), for which the shift operator is defined as

(5) $$S=\sum_{j=1}^{N}(|0,j\rangle \langle j,0|+\frac{1}{\sqrt{\left|N(j)\right|}}\sum_{k=1}^{N(j)}|1,j\rangle \langle k,1|),$$

where N(j) indicates the nearest neighbour set of node *j*.

When $\tilde{G}$ is a certain graph, the shift operator calculated via Equation (5) can be considered as a matrix composed of four block matrices with an *N*-dimension where the two diagonal matrices are the identity matrix and the matrix containing adjacency relation of network $\tilde{G}$, respectively. Other matrices are null matrices. In the shift operator *S*, this identity matrix and the matrix with adjacency relation are retained as components of the proposed simplified quantum walk model. This simple framework facilitates the advantages enumerated in the sequel.

The first effect of an identity matrix is equivalent to adding a self-loop to each node in $\tilde{G}$, while the effect of traceback generated by quantum superposition in a quantum walk will be effectively decreased. Traceback connotes that a particle oscillates or jumps repeatedly between the same pair of nodes. As exhibited in Figure 2b, for a quantum walk with a four-step walk, a possible traceback scenario where the particle jumps between node *j* and node *k* multiple times may occur. In this manner, this traceback will generate extensive redundant information, and such redundant information will disturb judgement regarding the accuracy of the observed probabilities in predicting the missing links.

Since the traceback is caused by quantum superposition, it cannot be eliminated by simply reducing the walk step. However, adoption of shift operator *S* presented in Equation (5) can effectively decrease the highlighted negative effects of traceback. As described in Figure 2c, a dangling node *j* has a unique walk direction if there is no self-loop over *j*, but the possibility of a particle staying at node *j* will be increased if the dangling node *j* has a self-loop because of a non-unique walk direction. This scenario occurs because the oscillations between a pair of nodes will be relatively decreased. In this manner, the shift operator *S* presented in Equation (5) can help curtail the negative effects generated by traceback. Moreover, the mechanism of self-loop can refer to a three-state quantum walk on one-dimensional lattice, where self-loop in the three-state quantum walks has been established to increase the probability of particle remaining at the initial node [30,31,32].

In the meantime, since the evolution operator is a composite of coin operator and shift operator, by combining Equation (5) and Equation (4), the evolution operator *U* of our proposed model can be defined as

(6) $$U=S\cdot (G^{C}⊗\hat{I}),$$

where $\hat{I}$ is a two-dimensional identity matrix that helps to maintain an identical dimension between *S* and $\left(G^{C}⊗\hat{I}\right)$. When the proposed quantum walk model is a *t*-step walk, the operator *U* is applied *t* times; hence, the evolution of network system $\tilde{G}$ after a *t*-step walk is defined as

(7) $$|\psi \left(t\right)\rangle =U^{t}|\psi \left(0\right)\rangle ,$$

where |ψ(t)〉 represents the state vector of the proposed model at time *t* (also interpreted as occurring evolution *t* times).

#### 2.2.3. Observation of Similarity between Two Nodes

After some finite-step walk, the observed probabilities on network $\tilde{G}$ can be used to estimate the similarity score of each link in $E^{U}$. For an arbitrary link $e\left(j,k\right)\in E^{U}$, its score $P_{e(j,k)}$ is equal to the possibility of e(j,k) being a missing link in network $\tilde{G}$. When the evolution operator is applied *t* times, the observation of similarity on e(j,k) can be interpreted as

(8) $$P_{e(j,k)}=\langle \hat{j}|U|\psi \left(t\right)\rangle =\langle \hat{j}|U^{t}|\psi \left(0\right)\rangle ,$$

where $\langle \hat{j}|$ is the conjugate transpose of $|\hat{j}\rangle$ and the evolution operator *U* takes up the state defined earlier in Equation (6). Considering two walk directions: leftward state |0〉 corresponding to walking along self-loop and rightward state |1〉 that corresponds to walking toward neighbours of node *j*, then $|\hat{j}\rangle$ is defined in the form presented in Equation (9).

(9) $$|\hat{j}\rangle =\frac{\left|\Gamma (j,k)\right|}{|E^{T}|}\sum_{c=0}^{1}|j\rangle ⊗|c\rangle ,$$

where $\frac{\left|\Gamma (j,k)\right|}{|E^{T}|}$ represents the ratio of the number of common neighbours between *j* and *k* to number of links in testing set $E^{T}$ (i.e., the number of links in $\tilde{G}$), and |j〉 denotes the orthogonal basis of node *j*. We note that, since for node *j*, the structural information of neighbours is contained in Equation (9), the orthogonal basis of node *k* need not be calculated again in Equation (8). The computed result of all links in $E^{U}$ in Equation (8) is then recorded as a similarity matrix, where each element is obtained by $P_{e(j,k)}$. Our walk step is set at 2, which attenuates the negative effects generated by traceback while retaining the quantum superposition in quantum walk.

**Figure 3:**
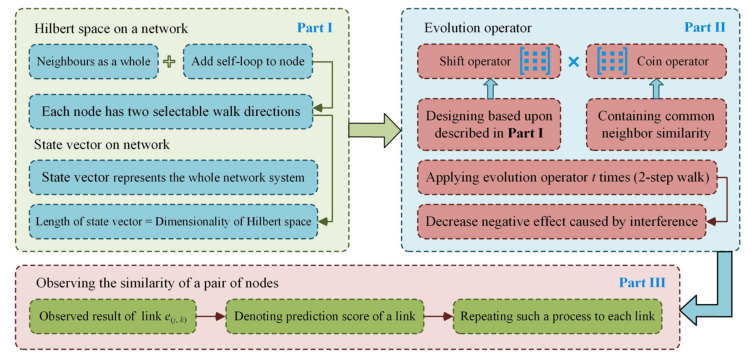
Architecture of the proposed simplified quantum walk model.

Combining the above designs and analysis, we obtain insight into the architecture of the proposed simplified quantum walk model, as presented in Figure 3. Consequently, compared to other quantum walk models for complex networks, our model differs in the following ways: (i) It avoids the constraint where an accumulated sum of observed probabilities of all nodes must equal to 1. This makes the observed result closes to the similarity expected between nodes, and more benefit structural characteristics will be captured by our proposed model. In fact, this property connotes the simplicity attributed to the proposed model. (ii) In other quantum walks on complex networks (e.g., the Fourier walk [22]), since a self-loop is equivalent to setting the diagonal element of the adjacency matrix as 1 and a node may have hundreds of neighbours in a complex network, it will cause the effect of a self-loop to be very weak. By contrast, in the evolution operator of our proposed model, the self-loop is denoted as an independent identity matrix, which can enhance the effect of particle staying at itself, i.e., relatively weakening the negative effect generated by traceback. (iii) Additionally, compared to existing quantum walk models, the proposed model decreased space complexity (total dimensionality of Hilbert space). Furthermore, based on the rule analysed in Section 2.2.2, the coding of the proposed model will consume less time, where key elements responsible are not available in other quantum walk models.

## 3. Prediction of Missing Links Using the Proposed Model

The experiments reported and discussed in this section were on nine complex networks; these networks cover multiple real-life seniors, including social relationships between members of a club, neural network of a human brain, co-occurrence relation of different nouns (words) in a novel, email interchanges among various individuals, air routes between US airports, and metabolic interaction between proteins and proteins. Key statistical characteristics of these complex networks are enumerated in Table 1, where *N* and *M* represent the amount of nodes and links, 〈k〉 and *d* are the average degree of networks and diameter of the networks, while *c* and ρ denote the clustering coefficient of network and associativity coefficient of the networks, respectively.

Furthermore, our proposed performance analysis is based on comparisons between the proposed model and fourteen algorithms on the selected complex networks. The fourteen algorithms include those on local neighbours-based algorithms [7,8,9,10,11,12], local or global paths-based algorithms [37,38], and random walk-based algorithms [15,16,39]. We note here that it has been observed that, in random walk-based algorithms, Neighbour Contribution (NC) offers significant advantage over traditional algorithms [15]. In the reported experiments, the walk step of such algorithms is preset to 3. The mathematical description of the fourteen algorithms is highlighted in Table 2, wherein $d_{j}$ represents degree of node *j*, *A* denotes the adjacency matrix of network $\tilde{G}$, *I* is an identity matrix, $l_{j,k}^{+}$ expresses an element in the Laplace matrix of network $\tilde{G}$, and $d_{max}$ is equivalent to the maximum degree in $\tilde{G}$.

### 3.1. Performance Analysis Using the AUC Index for Different Values of η

**Figure 4:**
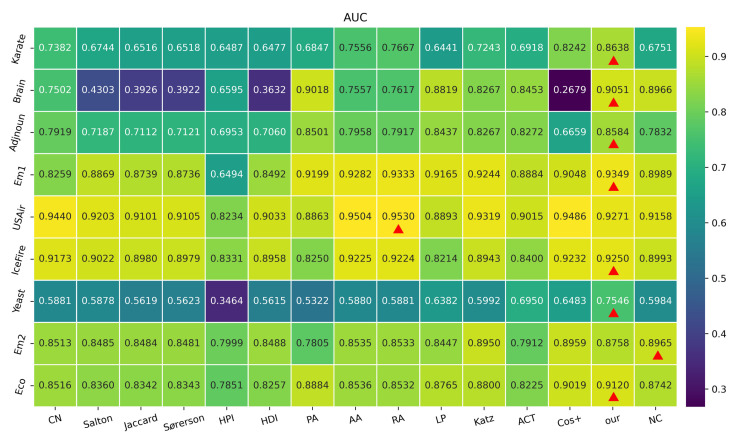
Outcomes of AUC index using various algorithms for η=0.9.

**Figure 5:**
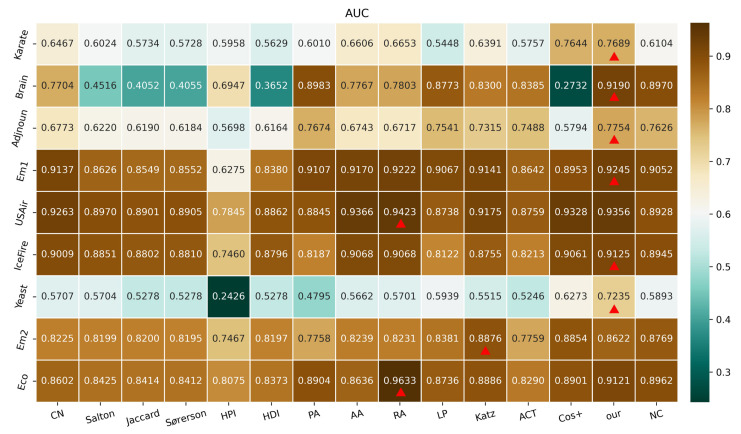
Outcomes of AUC index using various algorithms for η=0.8.

The sample times for *n* in Equation (1) are set to 672,400 in this experiment. This choice is motivated by observations that AUC has a guaranteed absolute error within the range of $10^{-3}$. For different values of η presented in Section 2.1, the evaluated values of AUC using various algorithms may change, since η represents the different size of training set $E^{T}$. In this experiment, η has default values of 0.9 and 0.8 for outcomes reported in Figure 4 and Figure 5, respectively. In these figures, each block denotes the AUC value using an algorithm on a complex network, where the highest AUC value in a network is marked with a red triangle, and the higher AUC values imply the better performance in the prediction of missing links.

As reported in Figure 4, on seven networks (i.e., Karate, Brain, Adjnoun, Em1, IceFire, Yeast, and Eco networks), the proposed model has the highest AUC values. As shown in Figure 5, on six networks (i.e., Karate, Brain, Adjnoun, Em1, IceFire, and Yeast networks), our model has the best performance according to AUC values. Concretely speaking, when our proposed model compared with different algorithms for link prediction, the following conclusions can be inferred. (i) Compared to the local neighbour-based algorithms (i.e., CN, Salton, Jaccard, Sorenson, HPI, and HDI), we note that, despite these competing algorithms sharing attributes of common neighbour information between arbitrary pair of nodes, like our proposed model, their performances do not match those recorded for our simplified quantum walk model using the AUC index. As a local path-based algorithm, the walk distance of the LP algorithm is equal to three, which is still no match for our proposed model (as presented in Figure 4 and Figure 5), whose walk step is only set at two. (ii) With regard to global algorithms, i.e., path-based (Katz) and random walk-based (ACT and Cos+) algorithms, the proposed simplified quantum walk model still records the highest accuracy on the whole, with the Katz algorithm better than our model in the Em2 network, as presented in Figure 5. (iii) Similarly, compared to the local random walk-based algorithm (i.e., NC), the performance by our model and the NC algorithm using the AUC index deserve special mention. Whereas NC outperforms the majority of the other competing algorithms, it only betters our proposed model in the Em2 network, as referenced with Figure 5. (iv) Additionally, even though the RA algorithm can be considered as a competitive method for all the comparison algorithms, its performance does not measure up with the proposed simplified model on the whole. Furthermore, when the performances between RA algorithm and our proposed model on the USAir network are considered, as shown in Figure 4, their AUC values are very approximate (i.e., 0.9423 and 0.9356).

To sum up, experiments based on the AUC index reported in this subsection indicate better performance for our proposed model in the prediction of missing links. Meanwhile, since there is randomness in the AUC value, further analysis of these outcomes using the precision index should be considered as reported in the next section.

### 3.2. Experiment on Precision Index under Various η

**Figure 6:**
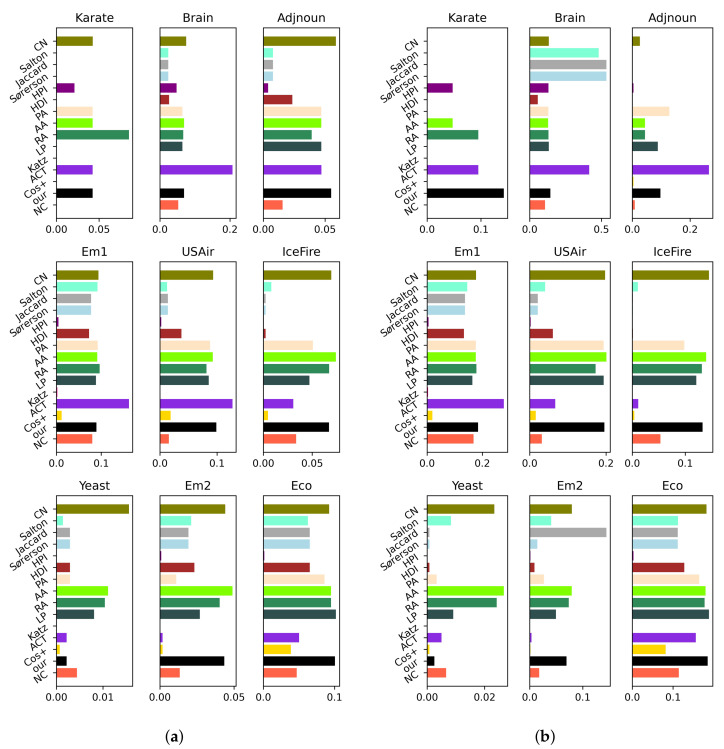
Experimental result on precision index using different algorithms. (**a**) precision results of various algorithms when η=0.9; (**b**) precision results using various algorithms when η=0.8.

Precision index is employed to further probe the performance of the proposed model in prediction of missing links for various algorithms, especially when their AUC values are approximately equal. In this experiment, the total number of links to be evaluated (i.e., *L*) defined in Equation (2) is preset as $\frac{1}{3}\left|E^{T}\right|$. The outcomes of the precision index are described in Figure 6, where higher values indicate better performance in link prediction. Figure 6a,b present outcomes for training probability parameter η set at 0.9 and 0.8, respectively.

The conclusions of this experiment are still analysed based on classifications of the comparison algorithms. (i) Despite CN records, the highest result in common neighbour-based algorithms, the precision results of other common neighbour-based algorithms are also good. However, our model approximately matches this best performance exhibited by the CN in the IceFire network and Eco network, as depicted in Figure 6a. This phenomenon implies that the common neighbour information is inherent to our proposed simplified quantum walk model, which is an important attribute in accurate prediction of missing links. (ii) Compared to global algorithms, the path-based algorithm (i.e., Katz) and random walk-based algorithms (i.e., ACT and Cos+), irrespective of the network used, our proposed simplified model outperforms the Katz and Cos+ algorithms in terms of a precision index. However, as reported in Figure 6a, we note that, even though the performance using the ACT algorithm is superior to the proposed model in Brain, Em1, and USAir networks, the proposed model records a better overall average performance. (iii) Similarly, as a random walk-based algorithm, NC slightly outperforms our proposed model in the Yeast network. By contrast, the proposed model is better than NC in other arbitrary networks, irrespective of the value of training probability parameter η. (iv) As mentioned earlier, the precision index is useful when probing the accuracy of different algorithms in the prediction of missing links. Considering the Eco network presented in Figure 5, we deduced that the RA algorithm has the highest AUC value (0.9633), while our proposal is a close second with an AUC value of 0.9121. However, as described in Figure 6b, the precision using our proposed model is better than what was recorded for the RA algorithm in the Eco network.

Looking at the two experimental scenarios that have been reported (AUC and precision indexes), it can be concluded that the precision result of the proposed model ranks in the top echelon of the comparison algorithms considered throughout the experiments, and our proposed model can predict the missing links in a complex network with high accuracy.

## 4. Conclusions and Future Work

A simplified quantum walk model on a complex network was proposed to predict missing links. The proposed model reduced the negative effect generated by traceback by adding a self-loop to each node and presetting the walk step to two. Furthermore, the observed probability at the end of the evolution operation is utilised to indicate the score of a missing link. Experiments to validate the efficacy of the proposed model indicated that it has better average performance in terms of the AUC than 14 competing algorithms. Furthermore, the precision index was used to probe outcomes with similarity results. Analysis of these outcomes suggested that the proposed model was better in missing link prediction than its competitors. These outcomes suggested promising results when the proposed model was deployed in different recommender systems.

There are many challenging issues based on this study. Firstly, the outcomes of the observed results between two nodes can be regarded as a similarity index between the nodes. It is a criterion in community detection of complex networks. Therefore, the proposed simplified quantum walk model can be employed to detect communities within a complex network. For example, since merging a pair of nodes with the highest similarity score as an independent community, then repeating such a procedure and aggregating these independent communities according to modularity function can result in a partitioning of the community detection, which is subsequently used in the proposed simplified model to obtain the similarity score. Secondly, by assuming that the hierarchy (importance) of missing links located in a network could influence the accuracy of an algorithm in link prediction; then, on the one hand, the accuracy using an algorithm may be very sensitive to the hierarchy (importance) of deleted links in the original network, while, on the other hand, the similarity information also contains the important details about links in a complex network (e.g., the significance and similarity of nodes can be simultaneously expressed through degree centrality). Consequently, to obtain a higher accuracy using the proposed model in link prediction, relations between the missing links and the accuracy of algorithms need to be further explored. Thirdly, since statistical complex networks were used in this study, their expressed information is incomplete, which makes prediction of missing links in the temporal (dynamic) complex networks a natural extension of the study since it is more practical.

## Figures and Tables

**Table 1:** Statistical characteristics of complex networks.

Networks	*N*	*M*	〈k〉	$d_{\max}$	*d*	*c*	ρ
Karate [33]	34	78	4.588	17	5	0.256	−0.475
Brain [33]	91	1989	43.714	248	3	1.304	−0.343
Adjnoun [34]	112	425	7.589	49	5	0.190	−0.129
Em1 [33]	167	3251	38.934	139	5	0.685	−0.295
Eco [33]	259	2942	19.560	108	6	0.395	0.018
USAir [33]	332	2126	12.807	139	6	0.749	−0.207
IceFire [35]	796	2823	81.982	122	9	0.209	−0.115
Em2 [33]	1133	5451	9.622	71	8	0.254	0.078
Yeast [36]	1870	2277	2.435	56	19	0.055	−0.161

**Table 2:** Overview of algorithms used in comparisons.

Algorithm (Indices)	Mathematical Expression
Common Neighbours [7] (CN)	$s_{j,k}^{CN}=\left|N\left(j\right)\cap N\left(k\right)\right|$
Salton Index [9]	$s_{j,k}^{Salton}=\frac{\left|N(j)\cap N(k)\right|}{\sqrt{d_{j}\cdot d_{k}}}$
Jaccard Index [8]	$s_{j,k}^{Jaccard}=\frac{\left|N(j)\cap N(k)\right|}{\left|N(j)\cup N(k)\right|}$
Sorenson Index [10]	$s_{j,k}^{Sørenson}=\frac{2|N(j)\cap N(k)|}{d_{j}+d_{k}}$
Hub Promoted Index [36] (HPI)	$s_{j,k}^{HPI}=\frac{\left|N(j)\cap N(k)\right|}{min(d_{j},d_{k})}$
Hub Depresses Index [37] (HDI)	$s_{j,k}^{HDI}=\frac{\left|N(j)\cap N(k)\right|}{max(d_{j},d_{k})}$
Preferential Attachment [11] (PA)	$s_{j,k}^{PA}=d_{j}\cdot d_{k}$
Admic-Adar [12] (AA)	$s_{j,k}^{AA}=\sum_{z\in N(j)\cap N(k)}\frac{1}{\log\left(k_{z}\right)}$
Resource Allocation [37] (RA)	$s_{j,k}^{RA}=\sum_{z\in N(j)\cap N(k)}\frac{1}{k_{z}}$
Local Path [37] (LP)	$S^{LP}=A^{2}+\alpha A^{3}$
Katz [38]	$S^{Katz}={\left(I-\alpha A\right)}^{-1}-I$
Average commute time [16] (ACT)	$S^{ACT}=A^{2}+\alpha A^{3}$
Random walk similarity by cosine [39] (Cos+)	$S_{j,k}^{ACT}=\frac{1}{l_{jj}^{+}+l_{kk}^{+}-2l_{jk}^{+}}$
Neighbour Contribution [15] (NC)	$s_{j,k}^{NC}\left(3\right)=\sum_{l=2}^{3}\left(\frac{\sum_{z\in N(j)}\frac{d_{z}}{d_{max}}}{2M}\pi_{jk}^{\left(l\right)}+\frac{\sum_{z\in N(k)}\frac{d_{z}}{d_{max}}}{2M}\pi_{kj}^{\left(l\right)}\right)$

## Data Availability

Not applicable.
